# A Hybrid EEG-Based Stress State Classification Model Using Multi-Domain Transfer Entropy and PCANet

**DOI:** 10.3390/brainsci14060595

**Published:** 2024-06-12

**Authors:** Yuefang Dong, Lin Xu, Jian Zheng, Dandan Wu, Huanli Li, Yongcong Shao, Guohua Shi, Weiwei Fu

**Affiliations:** 1 School of Biomedical Engineering (Suzhou), Division of Life Sciences and Medicine, University of Sciences and Technology of China, No.96, Jinzhai Road, Hefei 230026, China; dongyf@sibet.ac.cn (Y.D.); zhengj@sibet.ac.cn (J.Z.); fuww@sibet.ac.cn (W.F.); 2 Suzhou Institute of Biomedical Engineering and Technology, Chinese Academy of Sciences, No.88, Keling Road, Suzhou 215163, China; wudd@sibet.ac.cn; 3 School of Psychology, Beijing Sport University, Beijing 100084, China; 2023110049@bsu.edu.cn (L.X.); budeshao@bsu.edu.cn (Y.S.); 4 Luo Yang Institute of Science and Technology, No. 90, Wangcheng Avenue, Luoyang 471023, China; lihl@lit.edu.cn

**Keywords:** multi-domain, transfer entropy, 2D-PCANet, EEG, stress state

## Abstract

This paper proposes a new hybrid model for classifying stress states using EEG signals, combining multi-domain transfer entropy (TrEn) with a two-dimensional PCANet (2D-PCANet) approach. The aim is to create an automated system for identifying stress levels, which is crucial for early intervention and mental health management. A major challenge in this field lies in extracting meaningful emotional information from the complex patterns observed in EEG. Our model addresses this by initially applying independent component analysis (ICA) to purify the EEG signals, enhancing the clarity for further analysis. We then leverage the adaptability of the fractional Fourier transform (FrFT) to represent the EEG data in time, frequency, and time–frequency domains. This multi-domain representation allows for a more nuanced understanding of the brain’s activity in response to stress. The subsequent stage involves the deployment of a two-layer 2D-PCANet network designed to autonomously distill EEG features associated with stress. These features are then classified by a support vector machine (SVM) to determine the stress state. Moreover, stress induction and data acquisition experiments are designed. We employed two distinct tasks known to trigger stress responses. Other stress-inducing elements that enhance the stress response were included in the experimental design, such as time limits and performance feedback. The EEG data collected from 15 participants were retained. The proposed algorithm achieves an average accuracy of over 92% on this self-collected dataset, enabling stress state detection under different task-induced conditions.

## 1. Introduction

The stress state is an external emotional state that individuals undergo in response to both internal and external tense environmental stimuli. Stressful events are inevitable in life, and they trigger a series of reactions in the human body in unexpected tense situations, leading to stress pressure [1]. For instance, in a state of stress, the body exhibits physical reactions such as muscle tension, elevated blood pressure, and shortness of breath; emotional reactions such as anxiety, tension, and panic; and behavioral responses including avoidance, hesitation, and aggression [2]. While moderate stress pressure can enhance human activity efficiency and adaptive capabilities, excessive or prolonged stress states can have adverse effects on both physical and mental well-being and—in severe cases—pose a threat to human life. Therefore, effective identification of the human stress state is of significant importance for timely stress intervention and regulation to mitigate the associated risks to physical and mental health [3].

Traditional methods for stress assessment mainly rely on non-invasive approaches such as speech and facial expression analysis. Gupta et al. [4] proposed a driver stress detection method by analyzing facial expressions to monitor stress levels based on a hybrid deep convolutional neural network model. Meanwhile, Chu et al. [5] designed an automatic speech analysis program to evaluate stress burden and psychological health. However, these methods often focus on individual subjective tendencies or only consider the external manifestations of stress, resulting in limited evaluation mechanisms and significant individual variations in recognition accuracy. Recognizing and evaluating stress via physiological signals is of great significance since the changes in physiological features during stress are inherently difficult to conceal. Electroencephalogram (EEG) signals, which capture the overall electrical activity of brain neurons on the scalp or cortical surface, contain abundant physiological and psychological information. Consequently, they serve as an important objective basis for human stress state monitoring [6].

The analysis of EEG signals poses significant challenges due to their nonlinearity, non-stationarity, and susceptibility to interference. Currently, commonly used methods for EEG signal analysis include time domain, frequency domain, time–frequency domain analysis, and nonlinear dynamical analysis [7]. In recent years, EEG signals have been widely applied in stress recognition [8]. The literature review shows a major challenge in establishing a successful stress detection model: extracting emotion-related information from complex EEG signals. However, single-angle analyses in existing approaches reveal limited key features, making them ineffective in differentiating between different stress states.

To address this challenge, we propose a stress state recognition algorithm based on multi-domain transfer entropy (TrEn) and principal component analysis network (PCANet). Based on the flexibility of the fractional Fourier transform (FrFT) [9], we establish representations of TrEn in the time domain, frequency domain, and time–frequency domain, cleverly integrating temporal–spectral information and nonlinear information. Furthermore, to overcome the limitations of manual feature extraction, PCANet plays a crucial role in this approach through the automatic extraction of high-level emotional information from EEG signals. This achieves considerable performance gains with lower computational complexity, enhancing the reliability of the model in EEG classification. The present study focuses on exploring an effective stress state detection method through EEG. This study aims to contribute significantly to the field of affective computing and stress-related research, offering a robust and reliable method for stress state detection and analysis.

In summary, the main contributions of this work are listed as follows:

(1) By ingeniously leveraging the flexibility of fractional Fourier transform in varying the transform order, we achieved the representation of EEG signals in the time domain, frequency domain, and time–frequency domain. This established a multi-domain transfer entropy-based representation scheme for EEG signals, allowing the fusion of temporal–spectral information and better revealing the hidden details in EEG signals.
(2) We introduced deep PCANet, which automatically learns features from low-level and high-level EEG patterns within a supervised learning framework, instead of relying on manually selected features. This effectively avoids the subjectivity introduced by manual feature selection and enhances the model’s generalization capability and robustness.
(3) We designed a well-defined stress induction paradigm and collected EEG data from multiple participants. The proposed algorithm was validated on actual stress-inducing EEG data, and the experimental results demonstrated its effectiveness in the automatic detection of stress states based on EEG signals.

The structure of this paper is organized as follows. Section 2 presents the proposed algorithm framework. Section 3 explains the paradigm for EEG data collection. Section 4 analyzes the experimental results. Finally, Section 5 provides the discussion, and Section 6 presents the conclusion.

## 2. Data Collection Paradigms and Schemes

### 2.1. Stress Induction Paradigm

We collected and analyzed EEG data from 30 healthy male university students aged 18–23. Each participant performed two sets of game task experiments, employing two stress induction paradigms: the 2-back task [10] and the Balloon Analog Risk Task (BART) [11,12]. Our experimental design includes additional stress-inducing elements that complement the two tasks. For example, we have incorporated time constraints and performance feedback to further enhance the stress response. Previous studies [13,14] have demonstrated the effectiveness of the tests in inducing stress.

**Figure 1:**
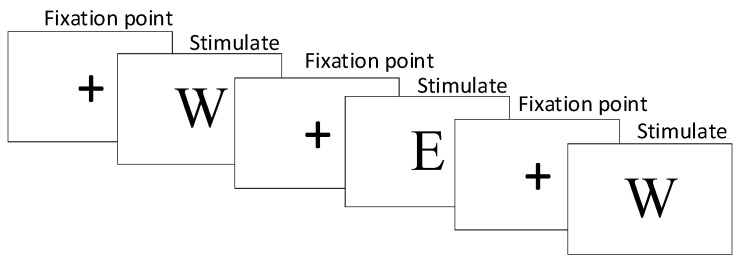
2-back task experimental paradigm.

For the 2-back paradigm, the experimental stimuli consisted of English letters such as W, R, E, Q, etc. The experiment began with the presentation of a fixation point “+” for 1000 ms, followed by the experimental stimuli for 2000 ms. Participants were instructed to compare the current stimulus with the one presented two trials back. They were required to press the F key if the stimuli were the same and the J key if they differed, with a time limit of 2000 ms to respond. The experiment consisted of a total of 50 trials, and the participants’ response results were recorded by the computer. After the experiment, the average accuracy rate of the participants was calculated. The specific task paradigm is shown in Figure 1.

**Figure 2:**
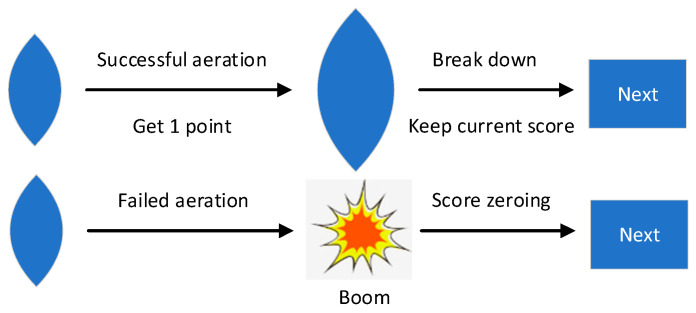
BART task experimental paradigm.

In the BART experimental paradigm, a deflated balloon appears at the center of the computer screen. With each press of the “inflate” key, the balloon expands by 0.3 cm, and the participant earns 1 point. However, each balloon randomly bursts between 1 and 30 inflations. During the inflation process, participants have the option to halt inflation at any point and transfer the current balloon earnings to their total earnings. In the event of a balloon bursting, the points accumulated for the “current balloon earnings” reset to zero. Participants can either continue inflating until the balloon bursts or press the “stop inflating” key to save the current earnings, thereby ending the trial. Subsequently, another balloon is presented to initiate a new trial, and this process continues until the end of the experimental session. The specific task paradigm is shown in Figure 2.

### 2.2. Stress Induction Protocol

The basic procedure of the laboratory simulated stress induction experiment is as follows:

(1) Experimental Setup: The experiment takes place in a quiet environment. Prior to the formal experiment, participants receive instructions regarding the experimental procedures and game controls. They are advised to maintain a calm state of mind. Before stress induction, their pre-stress EEG signals are recorded. After EEG data collection, participants complete the Subjective Anxiety Inventory (SAI) to assess their pre-stress emotional state.
(2) Stress Task Commencement: Participants engage in a game task under time constraints and negative feedback. During the game, instances of errors prompt real-time negative feedback such as “game failed” or “No points,” accompanied by a time penalty of a 10% reduction in the allocated task time. In order to increase the sense of stress, we will suddenly inform the participant before the task that the results of this task will be ranked and announced, and the final results will affect the final bonus amount.
(3) Stress Task Completion: After the stress task, participants again fill out the SAI to assess their post-stress emotional state.

**Figure 3:**
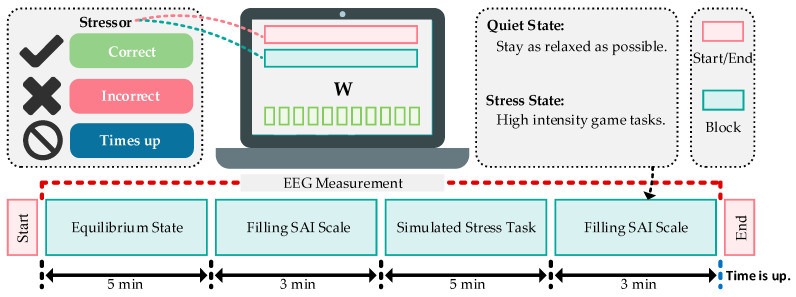
Experimental Procedure for EEG Data Collection. The “W” is one of the stimulus letters.

Each individual experiment lasts 16 min. The experimental procedure is depicted in Figure 3, with 5 min allocated for resting-state EEG data collection and another 5 min for stress-state EEG data collection. Each participant undergoes EEG data collection for the 2-back task and BART stress tasks on two separate days, with consistent data collection procedures.

### 2.3. Data Collection and Preprocessing

**Figure 4:**
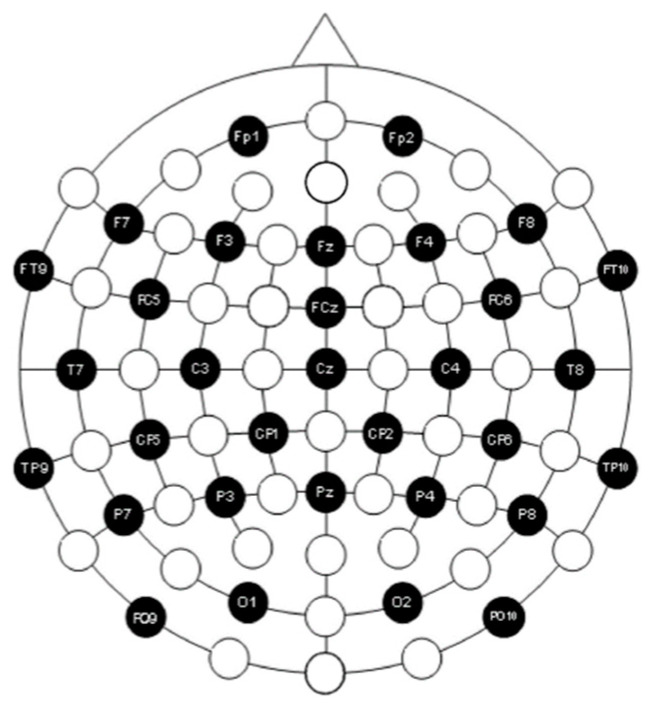
Electrodes used and their placement. The 32 electrodes used in this paper is marked in black, while the white ones are spare.

In this experiment, the EEG amplifier produced by Neuroscan was used for data collection. This equipment consists of 64 unipolar, 4 bipolar, and 2 high-level inputs, totaling 70 channels; while the sampling rate can reach 20,000 Hz. All channels use high-quality 24-bit A/D analog-to-digital chips to process data, which are transmitted to the computer via a USB 2.0 interface. We only applied a total of 32 scalp electrodes, including reference electrodes, positioned in accordance with the international 10–20 electrode placement system. The specific layout is shown in Figure 4. The sample frequency used for this experiment was set to 512 Hz. To ensure the validity and standardization of the experimental data, subjective rating scores before and after stressful scenario simulation assisted in the selection of EEG data. Samples with data loss due to uncontrollable reasons, those with no significant change in rating scores before and after the task, and samples where the game task was not successfully completed were excluded. Consequently, EEG data from 15 participants were retained for each of the two different stress tasks.

Before formal analysis of the EEG data related to stress states, it is necessary to perform simple preprocessing steps to standardize the data. Firstly, all EEG data undergo baseline correction by subtracting this average from each point in the EEG waveform. Subsequently, the Common Average Reference (CAR) method is applied to reduce the coupling effect between acquisition channels. Finally, the EEG signals are subjected to a band-pass filter and a 50 Hz notch filter to remove frequency components outside the range of 0.5–60 Hz and power line interference, respectively [15]. After simple data collation, the quality of EEG signals is preliminarily improved. In this paper, the EEG data are segmented using a sliding window of 2 s in length, without overlap between the windows.

## 3. Method

The stress state recognition algorithm proposed in this paper, based on multi-domain transfer entropy and PCANet, consists of five stages: independent component analysis (ICA) denoising, multi-domain FrFT decomposition, transfer entropy matrix generation, PCANet feature learning, and feature classification.

**Figure 5:**
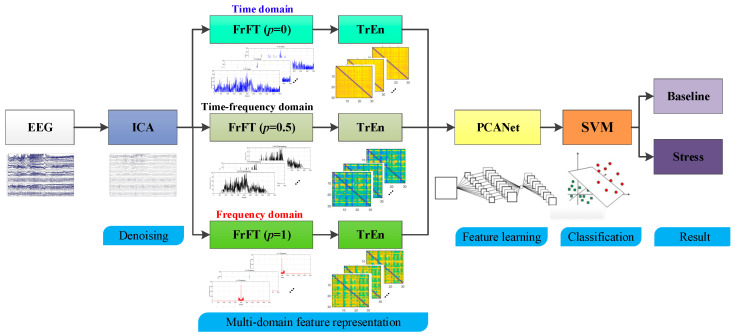
The architecture of the proposed algorithm. The main steps including denoising, muti-domain feature representation, feature learning, classification, and results.

The architecture of the proposed algorithm is illustrated in Figure 5. In the first stage, ICA is applied to eliminate ocular and muscular artifacts from the EEG signals. Subsequently, in the second stage, the EEG signals undergo projection into different domains by adjusting the rotation angle of the FrFT. In the third stage, the transfer entropy matrix of the EEG signals is computed under different decomposition perspectives across different domains. In the fourth stage, the 2D-PCANet, based on multi-domain transfer entropy and PCANet, is employed to automatically learn and discover features deeply rooted in the generated multi-domain transfer entropy matrix. This effectively captures the differences among various types of EEG signals. Finally, in the classification process, an SVM is used to assign a label to each extracted PCANet feature.

### 3.1. Independent Component Analysis (ICA)

ICA is used for array processing and data analysis. The aim is to recover unobserved signals from observed mixed signals under the assumption of signal independence [16,17]. The process begins with principal component analysis (PCA)—equivalent to singular value decomposition—followed by the recombination of these principal components to achieve temporal independence among the signals. Ideally, the number of collected mixed signals should equal or exceed the number of source signals [18]. Unlike other statistical methods, ICA measures independence using metrics such as mutual information, negative entropy, or correlation-based measures [19].

In the statistical model of ICA, considering m source signals and n observation points, the n observed values x are modeled as a linear combination of m sources:

(1) $$x_{i}=\sum_{j=1}^{m}a_{ij}s_{j}i=1,\ldots n$$

where $a_{i}$ are real numbers, and $s_{j}$ are statistically independent.

The variable $s_{j}$ represents latent variables that cannot be directly measured and are referred to as independent components. On the other hand, the observed results, $x_{i}$, are the only measurable observations, and they can be represented as a vector matrix:

(2) X=AS

where X is an n-dimensional observed random vector, A is the mixing matrix, and S is the matrix of independent sources, the goal is to estimate the original independent sources S from the observed values X [20].

Statistical independence between two source signals is essential in ICA. To ensure this, any two functions $h_{1}$ and $h_{2}$ must satisfy:

(3) $$E\left\{h_{1}\left(s_{1}\right),h_{2}\left(s_{2}\right)\right\}=E\left\{h_{1}\left(s_{1}\right)\right\}E\left\{h_{2}\left(s_{2}\right)\right\}$$

Thus, independence implies uncorrelatedness but not vice versa. A new observation matrix Z is obtained by whitening the original observed matrix X. Whitening involves subtracting the mean from X and applying principal component analysis to extract the orthogonal components that best represent the data, expressed as:

(4) Z=QX=QAS=BS

where Q is the whitening matrix, and B is an orthogonal matrix, which can be represented as:

(5) Z=BS

Then, it follows that that:

(6) $$E\left\{ZZ^{T}\right\}=E\left\{BSS^{T}B^{T}\right\}=BIB^{T}=I$$

where I is the identity matrix [21,22].

Based on Equation (2), the estimation of independent components is expressed as:

(7) Y=WX

where Y represents the estimated S, and W is the estimated demixing matrix, ideally given by $W=A^{-1}$. After whitening the original data, Equation (7) can be rewritten as:

(8) $$Y=WX=B^{T}Z=B^{T}QX=B^{T}QAS$$

where Y is the estimation of the source signals, and W is the corresponding demixing matrix, which can be represented as [23]:

(9) $$W=B^{T}Q$$

### 3.2. Fractional Fourier Transform (FrFT)

FrFT is one of the extensions of the Fourier transform that preserves its characteristics while allowing signal transformation into a domain between time and frequency [9]. As a result, FrFT finds wide applications in fields such as communication, radar, and encryption [24].

The pth order FrFT of a signal x(t) is represented as:

(10) $$\begin{array}{c} X_{p}\left(u\right)=F_{p}\left[x\left(t\right)\right]=\int_{-\infty }^{+\infty }x\left(t\right)K_{p}\left(u,t\right)dt \end{array}$$

Here, F[·] represents the FrFT operator, p is the order of the FrFT transformation, and $K_{p}(u,t)$ is the kernel function of the FrFT, given by:

(11) $$K_{p}\left(u,t\right)=\left\{\begin{array}{ll} \sqrt{\frac{1-\mathrm{jcot}\;\alpha }{2\pi }}exp\left(j\frac{u^{2}+t^{2}}{2}\cot\;\alpha -\frac{jut}{\sin\;\alpha }\right)\;\;\;\; & \alpha \neq n\pi \\ \delta (u-t)\;\;\;\; & \alpha =2n\pi \\ \delta (u+t)\;\;\;\; & \alpha =(2n\pm 1)\pi \end{array}\right.$$

where α=pπ/2 is the rotation angle. The flexibility of FrFT arises from the flexible rotation angle α. δ(t) represents the Dirac delta function [25].

From the above formulas, it can be observed that when p=1, i.e., α=π/2, the FrFT reduces to the ordinary Fourier transform:

(12) $$X_{1}\left(u\right)=F_{1}\left[x\left(t\right)\right]=\int_{-\infty }^{+\infty }x\left(t\right)K_{1}\left(u,t\right)dt=\frac{1}{\sqrt{2\pi }}\int_{-\infty }^{+\infty }\exp\;\left(-jut\right)x\left(t\right)dt$$

Moreover, for p=0, i.e., α=0, $X_{0}\left(u\right)=x\left(t\right)$, indicating no transformation applied to the signal. It is worth noting that α only appears in the trigonometric functions, making it periodic with a period of 2π. Therefore, p has a period of 4. It is sufficient to observe the signal’s behavior for α∈(−π,π] (or p∈(−2,2]) [26].

The following properties of the kernel function can be derived:

(13) $$K_{-\alpha }\left(u,t\right)=K_{\alpha }^{*}\left(u,t\right)$$

(14) $$\int_{-\infty }^{+\infty }K_{\alpha }\left(u,t\right)K_{\alpha }^{*}\left(u^{\prime },t\right)dt=\delta \left(u-u^{\prime }\right)$$

Hence, the inverse transformation of the FrFT can be obtained as:

(15) $$x\left(t\right)=F_{-p}\left[X_{p}\left(u\right)\right]=\int_{-\infty }^{+\infty }X_{p}\left(u\right)K_{-p}\left(u,t\right)du$$

**Figure 6:**
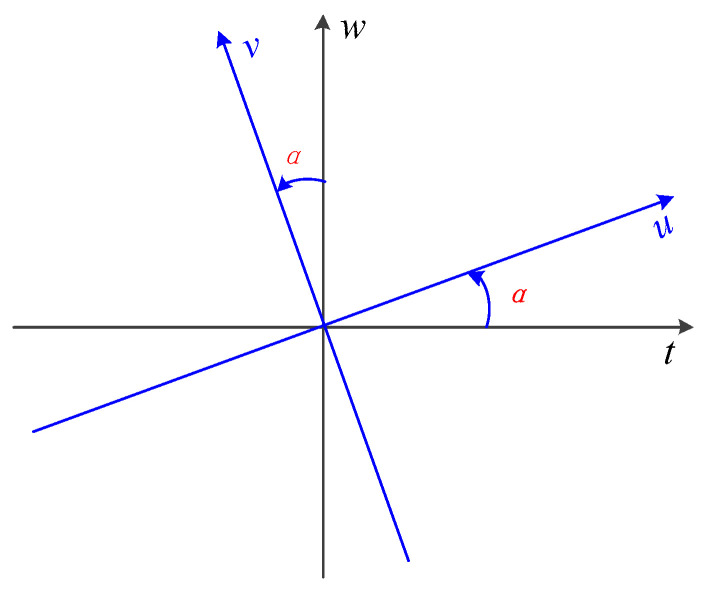
Time–frequency decomposition of FrFT. The α in red is the rotation angle of the time–frequency plane. The axis after rotation is marked in blue.

Figure 6 illustrates the time–frequency domain representation of the FrFT.

### 3.3. Transfer Entropy (TrEn)

Transfer entropy (TrEn), introduced by Schreiber, serves as an enhancement of information entropy. It analyzes information flow between two systems by examining the past values of one system alongside the current observations of another system. It quantifies the dependency between the two systems, with a higher value of transfer entropy indicating increased information transfer and a stronger correlation between the systems [27].

The specific definition of transfer entropy between two random processes, I and J, is as follows:

(16) $$T_{J\to I}=\sum p(i_{t+k},i_{t}^{\left(k\right)},j_{t}^{\left(k\right)})log\frac{p(i_{t+k}|i_{t}^{\left(k\right)},j_{t}^{\left(k\right)})}{p(i_{t+k}|i_{t}^{\left(k\right)})}$$

where $i_{t}$ and $j_{t}$ represent the observed values at time t. This expression can be simplified to:

(17) $$T_{J\to I}=H(i_{t+k}|i_{t})-H(i_{t+k}|i_{t},j_{t})$$

where, $H(i_{t+k}|i_{t})$ represents the conditional entropy, which measures the uncertainty of the variable at the current time given the knowledge of the past k time steps [28].

### 3.4. Principal Component Analysis Network (PCANet)

Principal Component Analysis Network (PCANet) is a convolutional neural network that builds upon PCA. It aims to address issues such as the need for a large number of training samples and the increasing number of parameters as research complexities increase. PCANet, proposed by Chan et al., 2014 [29], can be divided into three stages, and its network structure is as follows:

Assuming that the dataset comprises N training samples of size m×n, represented as:

(18) $$I=\{I_{i}{\}}_{i=1}^{N}$$

The filter size is set constant, $k_{1}\times k_{2}$.

First Stage: Block sampling is performed on each training sample, followed by mean subtraction (centering) on the sampled results. The sampled results of the *i*-th training sample can be represented as a matrix:

(19) $${\bar{X}}_{i}=[{\bar{x}}_{i,1},{\bar{x}}_{i,2},\ldots ,{\bar{x}}_{i,mn}],x_{i,1},x_{i,2},\ldots ,x_{i,mn}\in R^{k_{1}k_{2}}$$

The same mean subtraction process is applied to each sample in the dataset to yield a new matrix:

(20) $$X=\left[{\overset{¯}{X}}_{1},{\overset{¯}{X}}_{2},\ldots ,{\overset{¯}{X}}_{N}\right]\in R^{k_{1}k_{2}\times Nmn}$$

Assuming the *i*-th layer has $L_{i}$ filters, PCA is used to determine the principal component analysis matrix (standard orthogonal matrix) that minimizes the reconstruction error [30]:

(21) $$\underset{V\in R^{k_{1}k_{2}\times L_{1}}}{min}{∥X-VV^{T}X∥}_{F}^{2},s.t.V^{T}V=I_{L_{1}}$$

where $I_{L_{1}}$ is an $L_{1}\times L_{1}$ identity matrix. According to the PCA principle, $L_{1}$ is the eigenvector matrix of $XX^{T}$. Thus, the PCA filters are obtained by solving:

(22) $$W_{l}^{1}≐{mat}_{k_{1},k_{2}}\left(q_{l}\left(XX^{T}\right)\right)\in R^{k_{1}\times k_{2}},l=1,2,\ldots ,L_{1}$$

where mat is a function that maps a vector to a new space, and $q_{l}$ is a function that solves the parameter eigenvector matrix. It represents the eigenvectors of the matrix $XX^{T}$, which form the mapping matrix containing the main feature information of the samples [31]. The first-layer convolution is performed on the original dataset I. To ensure that the resulting $I_{i}^{l}$ in the first stage matches the original image $I_{i}$, zero-padding is applied to the edges of the original image since the mapping in the first stage may reduce the image size. The following formula represents the convolution process of the *i*-th image with $L_{1}$ feature maps:

(23) $$I_{i}^{l}≐I_{i}\times W_{l}^{1},i=1,2,\ldots ,L_{1}$$

The results of the first-layer convolution for the *i*-th image are concatenated to form a feature map:

(24) $$F_{i}^{1}≐{\left\{I_{i}\times W_{l}^{1}\right\}}_{l=1}^{L_{1}}=\left[I_{i}^{1},\ldots ,I_{i}^{L_{1}}\right]$$

Second Stage: Similar to the first stage, the second stage also requires block sampling and mean subtraction on each input feature map. Next, the *i*-th feature map $I_{i}^{l}$ is subjected to mean subtraction [32]:

(25) $${\overset{¯}{Y}}_{i}^{l}=\left[\begin{array}{c} {\bar{y}}_{i,l,1},{\bar{y}}_{i,l,2},\ldots ,{\bar{y}}_{i,l,mn} \end{array}\right]\in R^{k_{1}k_{2}\times m\hat{n}}$$

The feature maps produced by convolving the *l*-th convolutional kernel in the first layer undergo mean subtraction and can be uniformly represented as:

(26) $$Y^{l}=\left[\begin{array}{c} {\overset{¯}{Y}}_{1}^{l},{\overset{¯}{Y}}_{2}^{1},\ldots ,{\overset{¯}{Y}}_{N}^{l} \end{array}\right]\in R^{k_{1}k_{2}\times Nmn}$$

Therefore, the entire output of the first layer convolution after mean subtraction can be represented as:

(27) $$Y=\left[\begin{array}{c} Y^{1},Y^{2},\ldots ,Y^{L_{1}} \end{array}\right]\in R^{k_{1}k_{2}\times L_{1}Nmn}$$

Next, we solve for the convolutional kernels in the second stage. We set the kernel size to be $k_{1}\times k_{2}$ and the number of kernels to be $L_{2}$.

(28) $$W_{l}^{2}≐{mat}_{k_{1},k_{2}}\left(q_{l}\left(YY^{T}\right)\right)\in R^{k_{1}\times k_{2}},l=1,2,\ldots ,L_{2}$$

In the second stage, when convolving the $I_{i}^{l}$ feature maps generated in the first stage, $L_{2}$ corresponding feature maps are produced:

(29) $$O_{i}^{l}≐{\left\{I_{i}^{l}\times W_{l}^{2}\right\}}_{l=1}^{L_{2}}$$

After two rounds of PCA-based feature extraction on the *i*-th image, $L_{1}$ × $L_{2}$ corresponding feature maps are obtained in the second stage:

(30) $$O_{i}^{l}≐{\left\{I_{i}^{l}\times W_{l}^{2}\right\}}_{l=1}^{L_{2}},l=1,2,\ldots ,L_{1}$$

After the *i*-th image undergoes two layers of convolution, the final output results are concatenated, represented as:

(31) $$F_{i}^{2}={\left\{{\left\{I_{i}*W_{l}^{1}\right\}}_{l=1}^{L_{1}}\times W_{l}^{2}\right\}}_{l=1}^{L_{2}}=\left[O_{i}^{1},\ldots ,O_{i}^{L_{1}\times L_{2}}\right]$$

From the previous convolution iterations, it can be observed that PCANet exhibits strong scalability, making it easy to construct deep neural networks for feature extraction [33]. Here, only a brief introduction to PCANet with two iterations is provided.

Third Stage: Due to the exponential growth of feature dimensions with $L_{1}$ × $L_{2}$ feature maps generated in the second stage, the third stage employs hashing and histogram mapping methods to handle the feature maps and achieve discrete parameters and dimension reduction [34]. The specific steps are as follows:

First, binarization is performed on each feature matrix obtained from the second stage:

(32) $${\left\{H\left(I_{i}^{l}\times W_{l}^{2}\right)\right\}}_{l=1}^{L_{2}}$$

where H is a step function: if the original element of a pixel is greater than 0, the corresponding value in the new matrix is set to 1; otherwise, it is set to 0.

Next, hash encoding is performed on the processed feature matrices:

(33) $$T_{i}^{l}≐\sum_{l=1}^{L_{2}}2^{l-1}H\left(I_{i}^{l}\times W_{l}^{2}\right)$$

In other words, the binary representation of cap *L*2 is treated as a decimal number for each pixel, and the binarized feature maps into integer images.

Then, the encoded matrices are divided into blocks, with histogram statistics performed on each block

(34) $$f_{i}≐{\left[Bhist\left(T_{i}^{1}\right),\ldots ,Bhist\left(T_{i}^{L_{1}}\right)\right]}^{T}\in R^{\left(2^{L_{2}}\right)L_{1}B}$$

**Figure 7:**
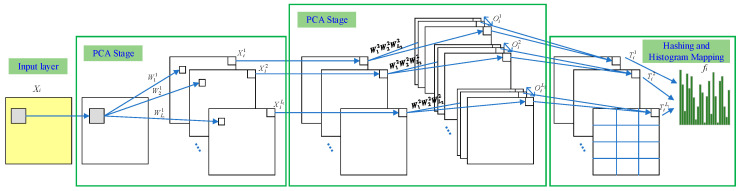
PCANet network architecture.

Therefore, in this third stage, the feature maps generated in the previous stage are processed using hashing and histogram mapping techniques to achieve discrete parameters and reduce the feature dimensions [35]. Figure 7 shows the PCANet structure.

### 3.5. Support Vector Machine (SVM)

**Figure 8:**
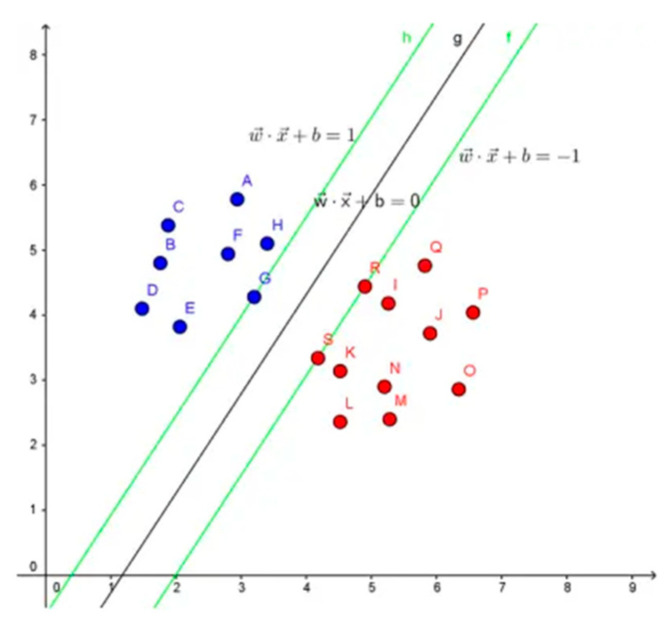
Illustration of SVM principle. The red and blue dots represent for two different kinds of samples. The green line represents for hyperplane.

SVM, a machine learning algorithm proposed by Chapelle et al., operates on the principle of structural risk minimization. Essentially, it maps data into a higher-dimensional feature space and locates a hyperplane in this projected space that maximizes the margin between classes [36]. Figure 8 illustrates the core principle of SVM. Its widespread use can be attributed to its outstanding problem-solving capacity related to small samples, nonlinearity, and high-dimensional recognition [37]. Due to the high dimensionality of the feature vectors, this study employs a linear kernel SVM. After hyperparameter fine-tuning, the penalty parameter *C* was set to 2. The value of *C* is changed from 1 to 10; the one that provided the best results is selected in this paper.

## 4. Experimental Results and Analysis

### 4.1. ICA Denoising Analysis

**Figure 9:**
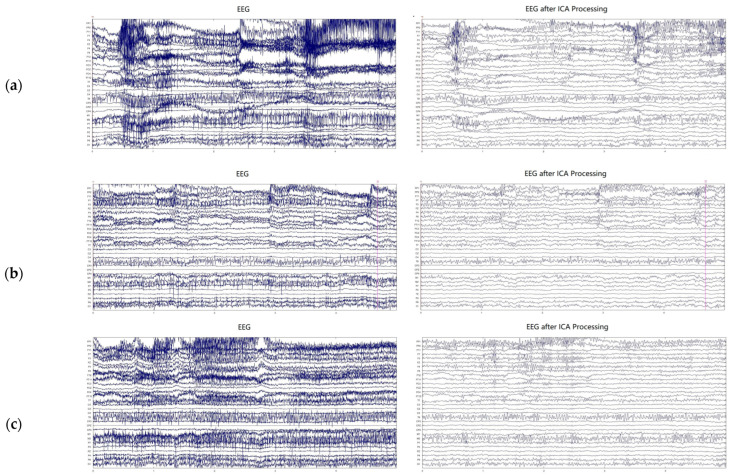
Comparison of original EEG signal and ICA denoised EEG; (**a**) Baseline, (**b**) 2-back, (**c**) BART.

In this study, the amplitude of the EEG signals was susceptible to artifacts such as eye movements and muscle activity. To address this issue and enhance signal quality, the filtered EEG signals were subjected to ICA. Figure 9 shows the original EEG signals alongside those after ICA denoising. From the figure, it can be observed that the EEG waveforms after ICA denoising appear smoother, with significantly reduced disturbances and a substantial reduction in sharp peaks. These observations indicate ICA effectively removes artifacts from the EEG signals.

### 4.2. Multi-Domain Representation Based on FrFT and TrEn

**Figure 10:**
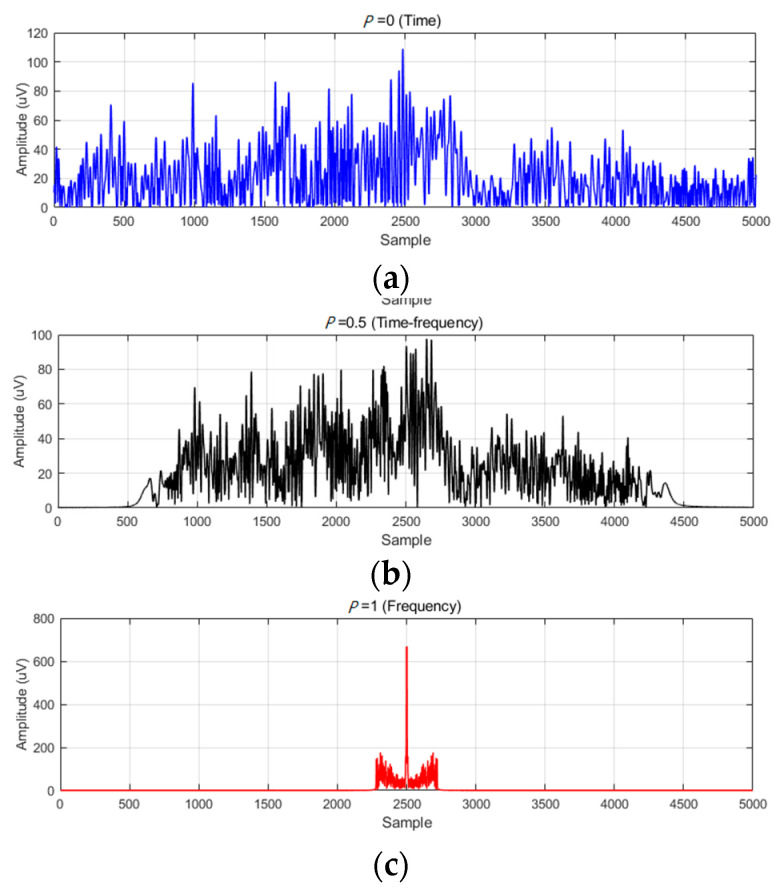
Absolute coefficients of the sample EEG after FrFT transformation; (**a**) *p* = 0 (time domain), (**b**) *p* = 0.5 (time–frequency domain), (**c**) *p* = 1 (frequency domain).

FrFT, a time–frequency analysis method, integrates both time and frequency domains, providing a unified representation in the time–frequency domain. Leveraging this characteristic, this study employs FrFT with different orders (*p*) to transform the EEG signals across the time, frequency, and time–frequency domains. Specifically, we set *p* to 0 (time domain), 0.5 (time-frequency domain), and 1 (frequency domain). To avoid the influence of complex values, we consider the absolute amplitude of the FrFT-transformed signals. Figure 10 illustrates the absolute coefficients of the sample EEG signals after multi-order FrFT transformation across different domains. As *p* changes, the EEG signals undergo transformations from the time domain to the time–frequency domain and then to the frequency domain, with the coefficients gradually becoming more compact and concentrated.

**Figure 11:**
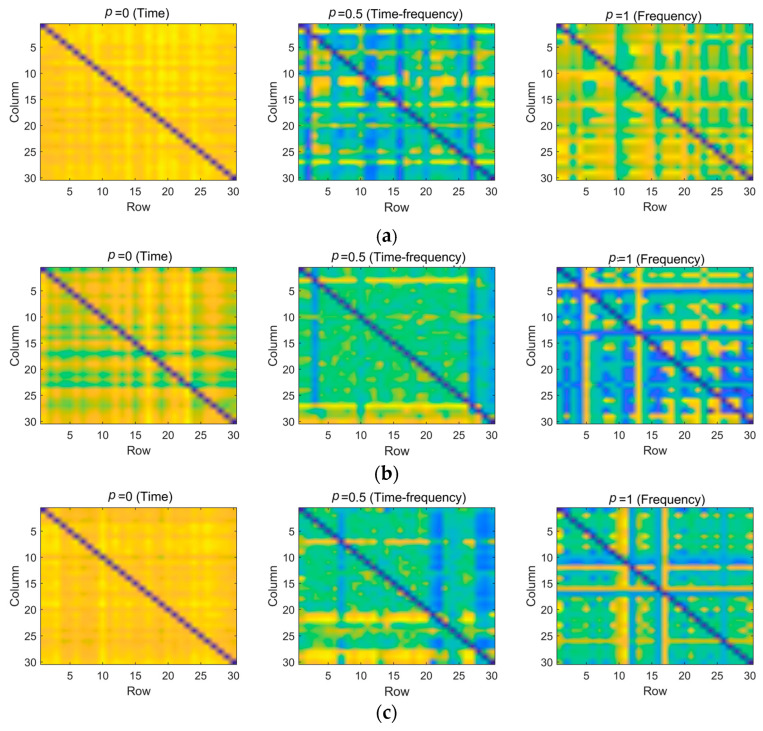
Connectivity matrix of TrEn for spatial connections in different domains; (**a**) Baseline, (**b**) 2-back, (**c**) BART.

After the multi-domain representation, TrEn is calculated to quantify the information transfer between different channels of the EEG, enabling the characterization of brain spatial information transfer across different domains. Figure 11 shows the TrEn matrices of the EEG signals in the time domain, frequency domain, and time–frequency domain under different states. These matrices depict variations in information transfer between electrodes across different decomposition domains, indicating the disclosure of distinct brain state information. Analysis of the spatial connectivity matrices of TrEn in the frequency domain and time–frequency domain reveals a significant decrease in the interaction strength of causal information among brain regions during stress tasks. Very strong connections between these brain regions are associated with emotion. This observation indicates that stress can impact the efficiency of brain functioning. Under stress, the brain becomes more active.

### 4.3. Classification Based on PCANet

The obtained multi-domain TrEn matrices are spatially integrated through cascading and fed into PCANet for automatic feature learning. After multiple training iterations, it was observed that the learning performance of PCANet does not further improve with an increase in layers. Therefore, a two-layer PCANet is sufficient to capture an adequate amount of stress state information. The optimal parameters for the PCANet structure are determined as shown in Table 1.

The obtained multi-domain transfer entropy-based PCANet features are then fed into an SVM classifier for stress state recognition. To be convinced, the 10-fold cross validation method is employed to evaluate the performance of the proposed method in detecting stress states in two tasks, namely BART and 2-back, separately. The results of the binary stress state EEG classification for 15 datasets in the two different tasks are shown in Table 2. From the results in Table 2, it is observed that the proposed algorithm achieves an average recognition accuracy of 92.14%, an average sensitivity of 94.25%, and an average specificity of 87.11% in the BART task. In the 2-back task, the proposed algorithm achieves an average recognition accuracy of 93.31%, an average sensitivity of 93.40%, and an average specificity of 93.22%. However, the stress recognition performance varies significantly among different subjects. For example, in the 2-back task, the highest recognition rate can reach 98.64%, while the lowest can be as low as 88.64%, with a difference of 10%. This variation is mainly due to the individual differences in how stress is manifested, as different subjects may exhibit varying levels of emotional response to the same stress task.

**Figure 12:**
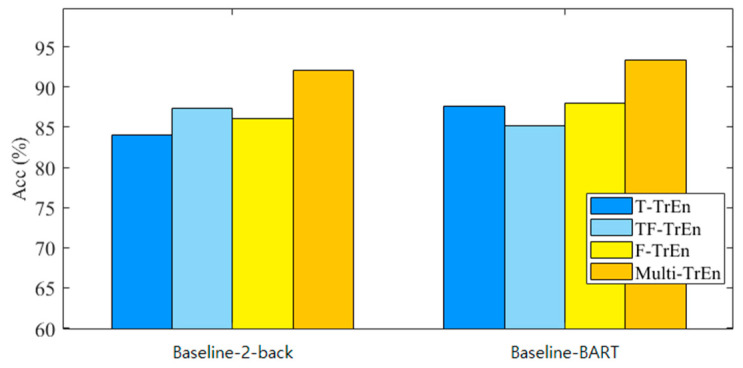
Performance comparison of TrEn in different single domains and multi-domain fusion. Specifically, T-TrEn, TF-TrEn, and F-TrEn represent TrEn representations in the single time domain, time–frequency domain, and frequency domain, respectively. Multi-TrEn represents the cascaded representation of the multi-domain fused TrEn.

To validate the effectiveness of the proposed multi-domain TrEn representation scheme, the study compared the recognition performance achieved by different single-domain TrEn representations. Figure 10 illustrates the overall recognition performance using different feature representation schemes. Specifically, T-TrEn, TF-TrEn, and F-TrEn represent TrEn representations in the single time domain, time-frequency domain, and frequency domain, respectively. On the other hand, Multi-TrEn represents the cascaded representation of the multi-domain fused TrEn. The proposed Multi-TrEn has achieved the best accuracies for both tasks. The results of other three methods are inferior to 90%. The results in Figure 12 demonstrate that the proposed multi-domain fusion strategy achieves information integration and complementarity, enhancing the learning efficiency of PCANet.

Furthermore, to further validate the performance of the proposed algorithm, this study conducted stress classification experiments in three states: Baseline, 2-Back, and BART. The results are shown in Table 3. The proposed algorithm achieved an average recognition accuracy of 91.81% for stress states in multi-stress tasks, along with an average Sen of 95.21% and an average Spe of 86.93%. The proposed multi-domain TrEn and PCANet models demonstrated significant advantages in both binary and ternary stress detection tasks, indicating the effectiveness and scalability of the algorithm.

## 5. Discussion

The present study introduces an automatic stress state recognition algorithm based on multi-domain transfer entropy and PCANet, aiming to identify an individual’s stress state through EEG signal analysis. The findings demonstrate that our proposed algorithm achieved an average accuracy rate exceeding 92% on a self-collected dataset, which is significant for the field of stress state detection.

Many researchers are working in this area. Sharma and his colleagues [38] utilized stationary wavelet transform to decompose EEG signals, extracted entropy-based features, and employed various evolutionary heuristic methods to optimize SVM. In their experiments, the Whale Optimization Algorithm optimized SVM and achieved better results. Tsai and his colleagues [39] collected EEG data from table tennis players, extracted features, and compared the performance of logistic regression, support vector machine, decision tree C4.5, classification and regression tree, random forest, and extreme gradient boosting (XGBoost) algorithms. Ultimately, XGBoost achieved an accuracy of 86.49% in classifying three stress states in a general model. Subhani and his colleagues [40] proposed a machine learning framework involving the analysis of EEG signals from stress participants. The results indicated an accuracy of 83.4% for multilevel stress recognition using this framework. The majority of studies have been conducted within the traditional single-domain framework, utilizing self-collected data. In this paper, we have proposed a novel method based on multi-domain TrEn and PCANet, which provides a new perspective for stress detection.

After ICA denoising processing, the quality of EEG signals was significantly enhanced, laying a solid foundation for subsequent feature extraction and state recognition. The EEG waveforms post-ICA denoising were smoother, indicating that this method effectively removed artifactual components from the signals, improving the signal-to-noise ratio. Leveraging the flexibility of the FrFT, we achieved multi-domain representations of EEG signals in the time domain, frequency domain, and time–frequency domain. The calculation of TrEn further revealed the state of information transfer between different brain regions, which is crucial for understanding the dynamic changes in the brain under stress (as shown in Figure 11). Experimental results indicate that as the stress task proceeds, the causal information interaction strength between subjects’ brain regions decreases, suggesting that stress states may affect the brain’s work efficiency. In terms of feature learning, the application of PCANet has shown its advantages in automatically extracting features from EEG signals. Within a supervised learning framework, PCANet can automatically learn both low-level and high-level features of EEG signals, avoiding the subjectivity brought about by manual feature selection, and enhancing the model’s generalization and robustness. SVM as a classifier also demonstrated good performance in this study. In summary, the automatic stress state recognition algorithm proposed in this study shows high accuracy and application potential in automatically detecting an individual’s stress state.

Although this study has achieved encouraging results, there is still room for improvement. One limitation in this paper is that the analysis of brain function was inadequate. Future research will pay more attention to brain function and brain networks under stress. We will expand the scope of experiments, including subjects of different ages, genders, and backgrounds, to enhance the model’s universality. Additionally, further diversifying stress-inducing paradigms and optimizing the algorithm structure may lead to further performance enhancement.

## 6. Conclusions

This paper proposes an automatic stress state recognition algorithm based on multi-domain TrEn and PCANet, providing a new approach for EEG-based emotion analysis. Based on the flexible transformation characteristics of the FrFT in the time–frequency domain, a multi-domain TrEn representation method is established, which integrates information from both time–frequency and spatial domains. This enables a multi-angle revelation of stress-related information. Furthermore, this study explores the application of PCANet—a deep feature learning model—in stress detection. This facilitates the automatic extraction of high-level and low-level features, eliminating the subjective influence of manual feature selection.

The proposed algorithm undergoes validation using authentic EEG data collected during stress-inducing tasks, showing recognition accuracies of over 92% in different stress-inducing tasks. This demonstrates its effectiveness and potential applications. Despite these promising outcomes, there is room for future research to further expand the experimental scope, diversify stress-inducing paradigms, brain function analysis, and optimize the algorithm structure.

## Figures and Tables

**Table 1:** PCANet structural parameters.

Parameter	Value	Parameter	Value
Layer numbers	2	Filter numbers (*L*1 × *L*2)	9 × 9
Input matrix size (*m* × *n*)	32 × 96	Block size	32 × 32
Patch size (*k*1 × *k*2)	3 × 3	Block overlap ratio	0.5

**Table 2:** Stress detection performance of the proposed method in different stress-inducing tasks. The final average results are in bold.

Group	BART			2-Back		
	Acc (%)	Sen (%)	Spe (%)	Acc (%)	Sen (%)	Spe (%)
1	94.76	95.35	93.66	90.33	93.10	87.66
2	94.65	96.60	91.00	93.22	90	96.33
3	88.37	89.82	85.66	90.50	88.27	92.66
4	93.48	96.60	87.66	98.64	97.93	99.33
5	93.48	95.71	89.33	92.88	96.20	89.66
6	94.53	97.5	89	94.06	97.93	90.33
7	88.72	93.75	89.33	92.71	95.51	90
8	92.55	86	88.33	95.59	96.20	95
9	90.69	91.96	88.33	94.40	93.79	95
10	93.13	95.53	88.66	90.84	90.68	91
11	90	93.39	83.66	92.54	92.41	92.66
12	91.97	96.07	84.33	98.30	97.93	98.66
13	89.53	93.57	82	92.71	89.31	96
14	93.72	95.35	90.67	94.23	92.41	96
15	92.55	96.60	85	88.64	89.31	88
**Ave**	**92.14**	**94.25**	**87.11**	**93.31**	**93.40**	**93.22**

Acc: Accuracy, Sen: Sensitivity, Spe: Specificity.

**Table 3:** Stress state classification for baseline, 2-Back, and BART modes. The final average results are in bold.

Groups	Acc (%)	Sen (%)	Spe (%)
1	94.80	98.95	88.33
2	98.27	99.47	97.22
3	88.70	97.76	91.58
4	93.21	99.32	96.32
5	91.71	95.41	86.19
6	91.89	98.35	82.86
7	90.85	96.59	81.43
8	90.35	94.47	87.00
9	98.87	91.53	91.67
10	87.13	95.41	82.67
11	94.35	95.18	83.33
12	86.96	85.18	85.00
13	86.00	92.59	81.00
14	91.04	95.53	82.67
15	93.04	92.47	86.67
**Ave**	**91.81**	**95.21**	**86.93**

Acc: Accuracy, Sen: Sensitivity, Spe: Specificity.

## Data Availability

The datasets generated for this study are available on request to corresponding authors. The data are not publicly available due to privacy and ethical reasons.
